# *Annona atemoya* Leaf Extract Improves Scopolamine-Induced Memory Impairment by Preventing Hippocampal Cholinergic Dysfunction and Neuronal Cell Death

**DOI:** 10.3390/ijms20143538

**Published:** 2019-07-19

**Authors:** Eunjin Sohn, Hye-Sun Lim, Yu Jin Kim, Bu-Yeo Kim, Soo-Jin Jeong

**Affiliations:** 1 Clinical Medicine Division, Korea Institute of Oriental Medicine, Daejeon 34054, Korea; 2 College of Pharmacy, Chungnam National University, Daejeon 34134, Korea

**Keywords:** memory impairment, scopolamine, acetylcholinesterase, *Annona atemoya* leaf, neurodegenerative diseases

## Abstract

We explored the preventative effect of *Annona atemoya* leaf (AAL) extract on memory impairment in a scopolamine (SCO)-induced cognitive deficit mouse model. Fifty-eight mice were randomly divided into six groups and orally treated with AAL extract at (50, 100, or 200 mg/kg) or tacrine (TAC) for 21 days. Memory deficits were induced by a single injection of 1 mg/kg SCO (i.p.) and memory improvement was evaluated by using behavioral tests such as the passive avoidance task and Y-maze test. The levels of cholinergic functions, neuronal cell death, reactive oxygen species, and protein expression related to hippocampal neurogenesis were examined by immunohistochemical staining and western blotting. The administration of AAL extract improved memory impairment according to increased spontaneous alternation in the Y-maze and step-through latency in passive avoidance test. AAL extract treatment increased the acetylcholine content, choline acetyltransferase, and acetylcholinesterase activity in the hippocampus of SCO-stimulated mice. In addition, AAL extract attenuated oxidative stress-induced neuronal cell death of hippocampal tissue. In terms of the regulatory mechanisms, AAL extract treatment reversed the SCO-induced decreases in the expression of Akt, phosphorylation of cAMP response element binding protein, and brain-derived neurotrophic factor. Our findings demonstrate that AAL extract has the ability to alleviate memory impairment through preventative effect on cholinergic system dysfunction and oxidative stress-related neuronal cell death in a SCO-induced memory deficit animal model. Overall, AAL may be a promising plant resource for the managing memory dysfunction due to neurodegenerative diseases, such as Alzheimer’s disease (AD).

## 1. Introduction

Dementia, which is characterized by aging-related memory and cognitive dysfunction and includes Alzheimer’s disease (AD), is becoming a more common public health problem as the lifespan of humans increases in modern industrial society [1,2]. Although the number of individuals with these diagnoses is gradually increasing, no curative medical treatment for dementia has been developed or approved by the US Food and Drug Administration (FDA), and drugs for AD provide only symptom relief [3]. Memory deficit is the most common symptom of neurodegeneration and results from neuronal dysfunction and neuronal loss in brain tissues, particularly in the hippocampal region [4]. It is generally accepted that AD is a complex neurodegenerative disease of multiple pathologies associated with central cholinergic system degeneration and oxidative stress [5]. Changes in cholinergic system activities are most strongly correlated with memory loss and cognitive impairments [6]. Additionally, cumulative evidences support the role of oxidative damage in the pathogenesis of AD, and neuronal degeneration in AD patients is associated with oxidative damage to all biomacromolecule types [7]. Various acetylcholinesterase (AChE) inhibitors, such as tacrine (TAC), donepezil, and rivastigmine, have been used as medications for AD patients [8]. However, all these drugs have limited effectiveness against AD due to their loss of efficacy and toxic side effects as the disease progresses [9,10,11]. There is currently no available satisfactory treatment to cure AD. Therefore, alternative and complementary therapies need to be developed for AD.

*Annona atemoya*, a fruitful plant of the annonacear family, is a hybrid of cherimoya (*A. cherimola*) and sugar apple (*A. squamosal*), and has temperature and humidity tolerance suitable for cultivation in tropical climates [12]. Moreover, economically, this genus is the most important of the family *Annonaceae* due to its edible fruits and medicinal properties [13,14,15]. Additionally, *A. atemoya* is cultivated and distributed in Jeju in Korea, Taiwan, and tropical areas such as Cuba and Jamaica [16,17]. Previous studies suggested that flavonoid isolated from *A. atemoya* fruit has anticancer activity conferred by reducing intracellular cAMP levels and inhibiting hepatocarcinoma cell proliferation [18,19]. Moreover, Yi et al. showed that an extract of *A. atemoya* seed exhibits antiangiogenic potential by regulating angiogenic pathways *in vitro* and *in vivo* [20]. Recently, other studies have reported that extracts from the leaves and stems of *A. atemoya* [21], as well as seven alkaloids from *A. atemoya* leaves identified by a series of spectrometric methods [22], exert antioxidant and antimicrobial activity.

However, there is no report on the biological activity of AAL for treating neurodegenerative conditions. In this study, we investigated whether ethanol extract from AAL exerts the preventative effect on scopolamine (SCO)-induced memory deficits in a mouse model. Additionally, the effects of AAL on neuronal dysfunction and neuronal loss were investigated to explain the underlying mechanisms.

## 2. Results

### 2.1. High-Performance Liquid Chromatography (HPLC) Determination of the Two Standard Compounds in AAL

**Figure 1:**
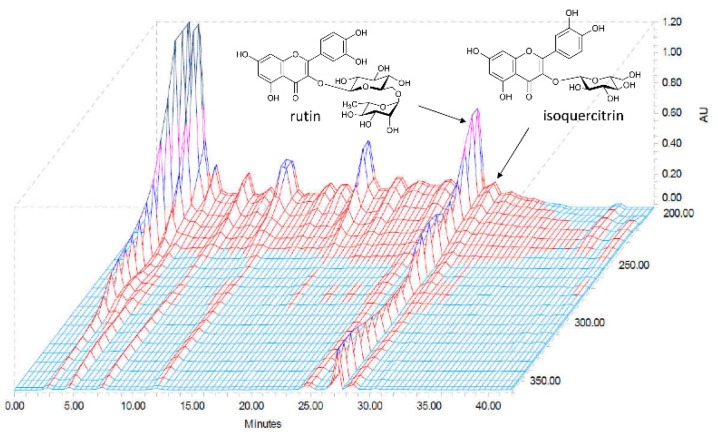
Three-dimensional HPLC chromatogram of *Annona atemoya* leaf (AAL) ethanol extract. A qualitative and quantitative analysis was performed by using HPLC. The compounds were identified by their retention times and UV spectrums relative to reference substances.

We used the optimized HPLC method to simultaneously determine the two standard compounds in AAL. We obtained good separation chromatograms using mobile phases consisting of 0.1% (*v*/*v*) aqueous trifluoroacetic acid (TFA) (A) and acetonitrile (B). The UV wavelengths for detecting compounds were 254 nm. Using the established HPLC methods, the two standard compounds were resolved within 30 min. The retention times of rutin and isoquercitrin were 27.18 and 28.47 min, respectively. Three-dimensional HPLC chromatogram of the AAL extract is presented in Figure 1. The amounts of the two major compounds rutin and isoquercitrin were 16.65 and 2.09 mg/g, respectively.

### 2.2. Effect of AAL Extract on Memory Deficit in SCO-Treated Mice

**Figure 2:**
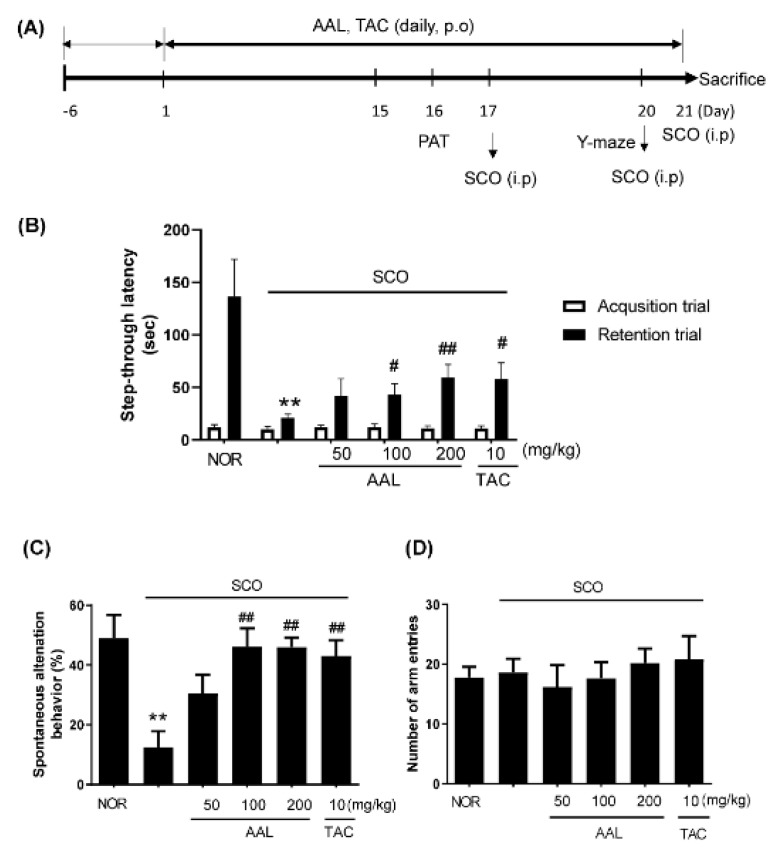
Effect of AAL extract on behavioral memory impairments in scopolamine-induced mice. Schematic description of the experimental timeline (**A**). For the passive avoidance test (PAT) (**B**), an acquisition trial was first performed and a retention trial was conducted for 300 s twenty-four hours after the acquisition trial. For the Y-maze test, the spontaneous alternation behavior (**C**) and the number of total arm entries were monitored during an 8-min session (**D**). Data are presented as the mean ± SEM (*n* = 8). ** *p* < 0.01 vs. NOR group, ^#^ *p* < 0.05 or ^##^ *p* < 0.01 vs. SCO group. NOR: normal control; SCO: scopolamine; AAL: *A. atemoya* leaf; TAC: tacrine.

To evaluate whether AAL extract enhances the recovery from cognitive deficit, SCO-induced cognitive deficit model mice performed PAT and Y-maze tests (Figure 2A). The SCO-treated group (SCO group) exhibited markedly reduced passive avoidance latency compared with the normal (NOR) group (*p* < 0.01). In contrast, AAL extract treatment significantly increased these latencies compared with the SCO group in a dose-dependent manner (Figure 2B). The deficit in spontaneous alternations in the Y-maze test was significantly reversed in a dose-dependent manner in the SCO and AAL extract-treated group (AAL group) compared with the SCO-injected group (Figure 2C). The SCO and TAC-treated group (TAC group) also exhibited a marked attenuation of SCO-induced memory deficit. TAC treatment was used as a positive control. No significant difference was observed in the number of arm entries among the experimental mouse groups (Figure 2D).

### 2.3. Effect of AAL Extract on Neuronal Cell Loss in SCO-Treated Mice

**Figure 3:**
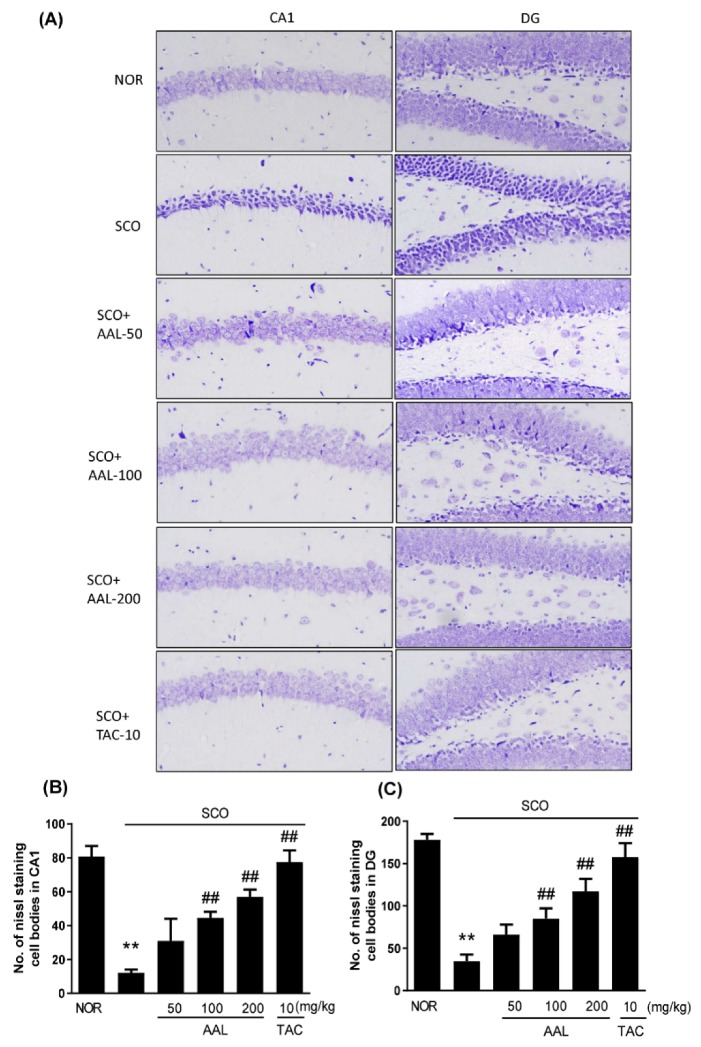
Effect of AAL extract on the morphological damage to neurons in scopolamine-induced cognitive deficit mouse brains. Sections of the hippocampus, including the CA1 and DG regions, were prepared for Nissl staining using cresyl violet solution. Representative photomicrographs are shown at magnifications of ×400 (**A**). The graph shows the number (No.) of Nissl stained cells in the CA1 and the DG (**B** and **C**, respectively). CA: Cornu Ammonis; DG: dentate gyrus, Data are presented as the mean ± SEM (*n* = 3). ** *p* < 0.01 vs. NOR group, ^##^ *p* < 0.01 vs. SCO group. NOR: normal control; SCO: scopolamine; AAL: *A. atemoya leaf*; TAC: tacrine.

The neuroprotective effect of AAL extract in SCO-mediated memory deficit mice was identified using cresyl violet staining in the hippocampus of mouse brains. As shown in Figure 3, the SCO group displayed marked neuronal damage and nucleus shrinkage, or staining in different shades in the Cornu Ammonis (CA) 1 and dentate gyrus (DG) regions of the hippocampus compared with the NOR group (Figure 3A). After treatment with AAL extract or TAC, the SCO-induced neuron damage was fairly well prevented in the hippocampus (Figure 3B,C).

### 2.4. Effect of AAL Extract on Cholinergic System and Oxidative Stress in SCO-Treated Mice

**Figure 4:**
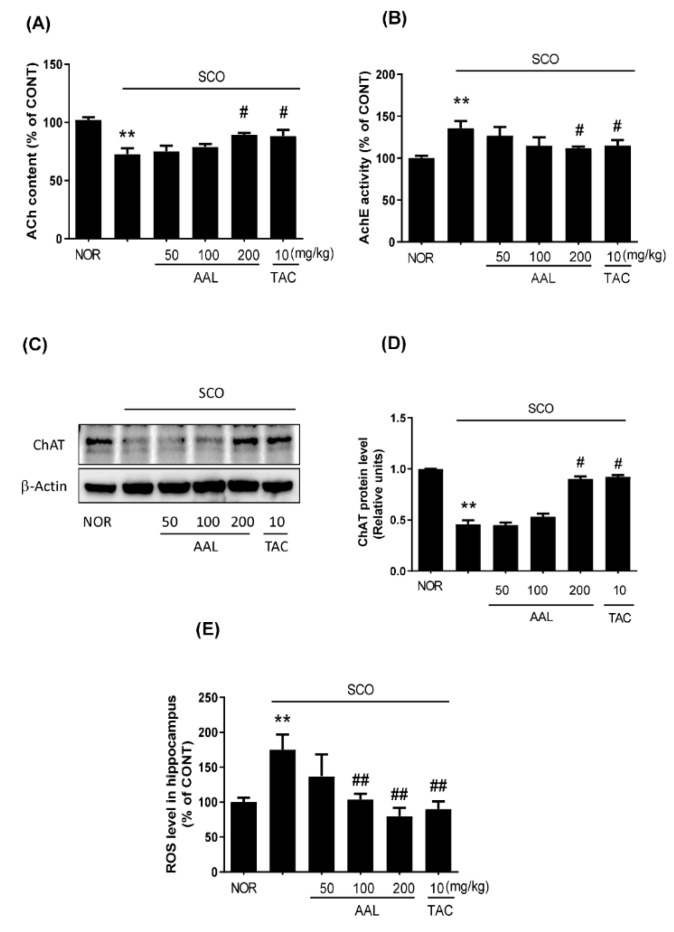
Effect of AAL extract on cholinergic system dysfunction and ROS production in scopolamine-induced cognitive deficit mouse brains. ACh content (**A**) and AChE activity (**B**) were measured in the hippocampus using an ACh and AChE activity assay kit (US Biomax Inc., Rockville, MD, USA). Hippocampus tissue was lysed and subjected to Western blotting with anti-ChAT antibody (**C**). Expression levels were normalized to β-actin. Bar graphs represent the relative band intensities compared with NOR (**D**). ROS levels in hippocampal tissue were measured using an ROS/RNS assay kit (Cell Biolabs, Inc., Sandiego, CA, USA). The fluorescence intensities corresponding to the ROS levels in the hippocampus are represented as a percentage of that in the NOR (**E**). Data are presented as the mean ± SEM (*n* = 5). ** *p* < 0.01 vs. NOR group, ^#^ *p* < 0.05 or ^##^ *p* < 0.01 vs. SCO group. NOR: normal control; SCO: scopolamine; AAL: *A. atemoya* leaf; TAC: tacrine.

Acetylcholine (ACh), a major excitatory neurotransmitter in neurons, is important for the formation of retention of existing memory [23]. To further elucidate the potential mechanisms of AAL extract in improving memory induced by SCO, the levels of multifarious biochemical factors and proteins associated with the cholinergic system and oxidative stress were investigated. As shown in Figure 4, only treatment with SCO led to a remarkable decrease in ACh content and an increase in AChE activity in the hippocampus. In contrast, the AAL extract or TAC treatment group significantly reversed the effects of SCO on ACh content and AChE activity (Figure 4A,B). Consistently, the SCO group significantly decreased choline acetyltransferase (ChAT) protein expression, whereas AAL extract or TAC treatment prevented the SCO-mediated decrease in ChAT expression in the hippocampus (Figure 4C,D). Oxidative stress is related to the cognitive deficits observed in the SCO-induced cognitive deficit mouse model [24]. The SCO group exhibited significantly increased ROS levels compared with the NOR group (Figure 4E). In contrast, AAL extract treatment attenuated the SCO-induced increase in reactive oxygen species (ROS) levels in the hippocampus. The TAC group also exhibited decreased ROS levels in SCO-induced memory deficit mice. These results suggested that AAL extract could protect against SCO-induced dysfunction of the cholinergic system and oxidative stress in brain tissue.

### 2.5. Effect of AAL Extract on Apoptosis in SCO-Treated Mice

**Figure 5:**
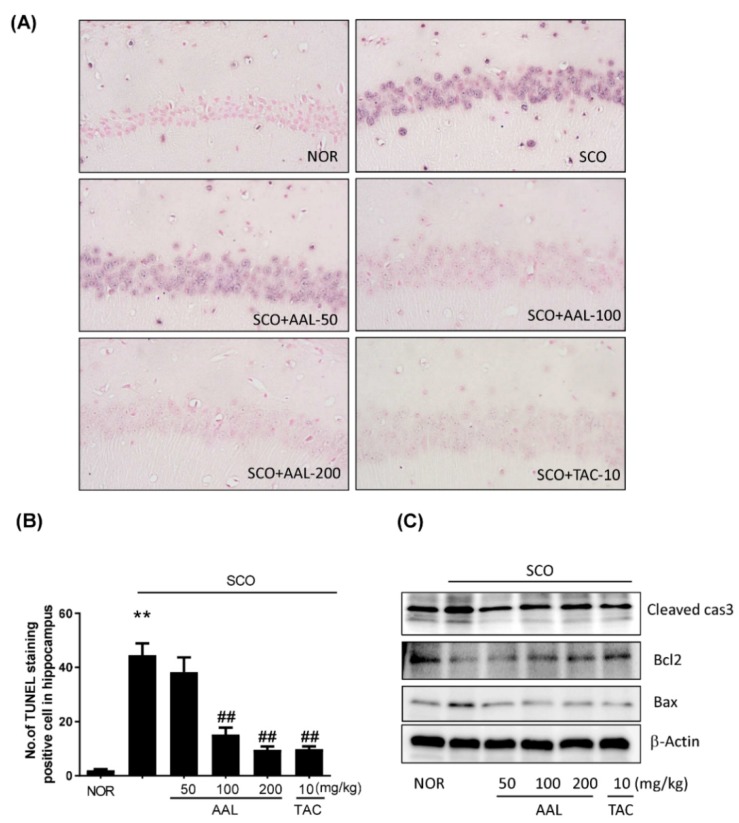
Effect of AAL extract on the expression of apoptosis-related proteins in scopolamine-induced cognitive deficit mouse brains. Representative photomicrographs were taken at magnifications of ×400 (**A**). Apoptotic cells were visualized with nitroblue tetrazolium and 5-bromo-4-chloro-3-indolylphosphate (NBT/BCIP-AP, purple). Counterstaining for nuclei was performed with Fast-red. Bar graphs represent a quantitative analysis of the apoptotic signals in histological sections of the hippocampus (**B**). Data are presented as the mean ± SEM (*n* = 3). ** *p* < 0.01 vs. NOR group, ^##^ *p* < 0.01 vs. SCO group. Hippocampal tissues were lysed and subjected to Western blotting with anti-Bax, Bcl2 and cleaved caspase-3 antibodies (**C**). Expression levels were normalized to β-actin. NOR: normal control; SCO: scopolamine; AAL: *A. atemoya* leaf; TAC: tacrine.

Apoptosis has been reported to be related with mechanisms of oxidative stress and central cholinergic system dysfunction [25]. Thus, neuronal apoptosis in the hippocampus of brain tissue was determined by Western blotting and TUNEL staining. As shown in Figure 5A,B, apoptotic cells were stained purple in the hippocampal region in the SCO group. AAL extract or TAC treatment resulted in a dramatic decrease in the number of apoptotic cells in the hippocampal region compared with the number observed in the SCO group. The relative protein levels of cleaved caspase-3 and Bax were increased in the SCO group compared with the NOR group. The relative protein level of Bcl2 was lower in the SCO group than in the NOR group. In contrast, the AAL extract and TAC groups exhibited significant preventative effects on apoptotic activation in the SCO-induced cognitive deficit mouse brain. These results suggest that treatment with AAL extract markedly attenuated apoptosis in the hippocampus region of SCO-induced cognitive deficit mice (Figure 5C).

### 2.6. Effect of AAL Extract on SCO-Attenuated Expression of BDNF and Phosphorylation of CREB and Akt in the Hippocampus

**Figure 6:**
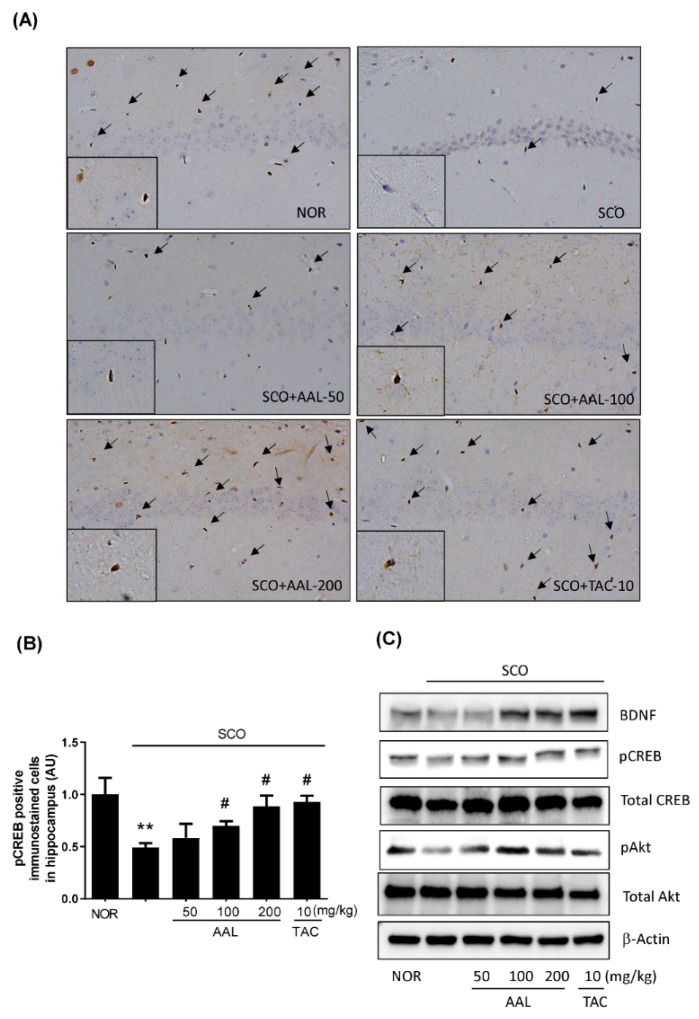
Effect of AAL extract on the levels of hippocampal BDNF, pCREB, and pAkt proteins in scopolamine-induced cognitive deficit mouse brains. Representative images show DAB immunostaining for phospho-CREB (pCREB) in the hippocampal region (**A**). The immunostained positive cells were calculated in brain tissue sections from each mouse (**B**). The graphs show the number of pCREB-positive cells in the hippocampal region. Data are presented as the mean ± SEM (*n* = 3). ** *p* < 0.01 vs. NOR group, ^#^ *p* < 0.05 vs. SCO group. Hippocampus tissues were lysed and subjected to Western blotting with anti-BDNF, phospho-CREB, total CREB, phospho-Akt, and total Akt antibodies (**C**). The protein levels were normalized to total CREB, total Akt, and β-actin, respectively. NOR: normal control; SCO: scopolamine; AAL: *A. atemoya* leaf; TAC: tacrine.

Brain Derived Neurotrophic Factor (BDNF) and phosphorylated cAMP response element binding protein (pCREB) are critical factors associated with learning and memory formation, and activation of CREB transcriptional activity regulates BDNF expression to induce learning and memory function [26]. The effects of AAL extract on the expression of BDNF and phosphorylation of CREB in mouse brain tissues were examined by immunohistochemistry and Western blot analysis. As shown in Figure 6A,B, the SCO group showed the reduced pCREB and BDNF levels in the hippocampus. Meanwhile, AAL extract treatment markedly prevented the SCO-induced decrease in pCREB and BDNF levels (*p* < 0.01). We also examined Akt activation to investigate the potential mechanisms by which AAL extract may promote hippocampal neurogenesis and protect against SCO-induced memory deficits. Our results showed that AAL extract protected against SCO-mediated Akt inactivation in hippocampal tissues (Figure 6C). These results suggest that AAL extract may promote memory function through a mechanisms associated with the activation of neurogenic factors BDNF/CREB and survival protein Akt in the SCO-induced memory deficit mouse model.

## 3. Discussion

Cumulative evidence has suggested that cognitive dysfunction in AD is the result of impaired neurogenesis in the hippocampus of the adult brain, and it is crucial to identify novel drugs that can be used for the treatment of AD [27,28]. The cholinergic function is reduced in the forebrain, especially in the hippocampus, and a change in AChE activity in the hippocampus is usually a biomarker for diagnosing AD [29]. SCO impairs memory in rodents and humans, which interferes with the cholinergic system, including ACh, and causes oxidative stress, leading to cognitive dysfunction [30,31,32]. Thus, SCO-induced cognitive impairment is a valid model to investigate the anti-AD effects of novel drugs. In the present study, we investigated the effects of AAL extract in a SCO-induced memory deficit mouse model. The results showed that AAL extract treatment significantly improved memory dysfunction by ameliorating cholinergic system impairment and oxidative stress in SCO-induced cognitive deficit mice. In addition, AAL extract could have a neuroprotective effect against SCO-induced apoptosis in the hippocampus.

AD is highly related to cholinergic system impairment and intracellular oxidative stress [7,29]. Hippocampal cholinergic dysfunction has been identified in neurodegenerative diseases that are characterized by memory impairment, including AD, and in animal experiments [33,34]. Previous studies reported that mice receiving SCO alone showed a marked decrease in the cholinergic system reactivity, as indicated by decreased ACh level and increased AChE activity along with decreased ChAT activity, resulting in cognitive deficiency and neuronal cell death in the hippocampus [35,36]. It has been established that SCO induces memory impairment associated with dysfunction of cholinergic neurotransmission as well as increases processes connected with oxidative stress in the brain [37]. An imbalance between ROS production and antioxidant capacity is associated with numerous diseases, including neurodegenerative diseases [38]. ROS play a critical role in neuron loss and can cause extensive damage to proteins and DNA, leading to changes in the structures and functions of neural cells in the brain [39]. Apoptosis is considered to be one of the main causes of neurodegeneration, and ROS can directly lead to apoptosis responses. Of the caspase family, caspase-3 is the terminal executing enzyme in apoptosis. Caspase-3 can disrupt the structural protein of cells and lead to apoptosis. In contrast to the antiapoptotic protein Bcl2, the proapoptotic Bax protein is associated with the activation of apoptosis [40]. Thus, protective effects against apoptosis and oxidative damage in neurodegenerative diseases are important factors to consider in the discovery of new therapeutic drugs.

In the present study, we used the Y-maze test and PAT to measure memory deficits in SCO-treated mice. The results showed that AAL extract treatment significantly improved spontaneous alternation in the Y-maze test and the step-through latency in the retention trial of the PAT in SCO-induced cognitive deficit mice. TAC (positive control) could also effectively ameliorate these changes in SCO-induced cognitive deficit mice and improve their learning and memory. Moreover, AAL extract effectively attenuated the SCO-induced dysfunction of the cholinergic system and neuronal cell damage in the hippocampus in the brain, corresponding to a preventative effect against memory impairment. We found that AAL extract dramatically increased the scavenging rates against ABTS and DPPH radicals, indicating antioxidant activity of AAL (data not shown, unpublished data). In addition, this study showed that AAL extract significantly reduced ROS production in hippocampal tissues of SCO-induced cognitive deficit mice. TUNEL staining also demonstrated that AAL extract significantly ameliorated neuronal apoptosis, including a reduction in cleaved caspase-3 and Bax protein expression in the hippocampus of SCO-induced cognitive deficit mice. These results suggested that the protective effect of AAL extract against SCO-induced memory deficit is related to inhibit the damage caused by ROS and the subsequent apoptosis. Therefore, in this study suggests that AAL extract might ameliorate memory impairment through the anticholinergic system and by preventing oxidative stress and neuronal cell apoptosis.

Many studies have shown that the signaling pathways involving BDNF/pCREB and Akt play crucial roles in the modulation of hippocampal neurogenesis in memory-impaired rodent models and in patients with dementia [41,42,43]. Activation of CREB transcriptional activity regulates BDNF expression to induce cognitive function. BDNF overexpression increases Akt activation and stimulates pCREB in neuroprotection against brain pathology [44,45]. In agreement with these findings, we confirmed that SCO treatment markedly decreased the expression of BDNF, and levels of pCREB and pAkt by Western blot analysis. Consistently, SCO administration decreased the number of pCREB-positive cells in the hippocampal region by immunostaining. In contrast, treatment with AAL extract effectively prevented these SCO-induced reductions in BDNF/pCREB and pAkt. These findings indicate that AAL exerts memory-enhancing effects through regulation of the BDNF/pCREB and Akt pathways.

HPLC analysis confirmed that AAL has two major components rutin and isoquercitrin, which constitute 16.65 and 2.09 mg/g, respectively. In previous studies, rutin showed potential neuroprotective effects against cerebral injury induced by ischemic reperfusion by ameliorating neurological impairments and oxidative damage [46,47]. Moreover, rutin alleviated AD-type streptozotocin-induced neurodegeneration and the associated cognitive impairment in a working memory animal model [48]. Rutin has the potential to prevent short- and long-term memory deficits; the use of rutin would contribute to increase plant-derived products for use as medicines to treat neurodegenerative diseases. Isoquercitrin is one of the main flavonoid glycosides and has been reported to have beneficial effects against oxidative stress, diabetes, and cancer and act as an anti-inflammatory agent [49,50,51,52].

Ip PS et al. recently suggested that complementary and alternative medicines could be potential sources of new drugs for the prevention of neurodegenerative diseases [53]. Earlier reports revealed that dietary intake of rich polyphenolic vegetables and fruits delayed the onset of dementia associated with AD [54,55]. Although *N*-methyl D-aspartate receptor antagonists and cholinesterase inhibitors have been widely used for treating the symptoms of AD, these drugs have not shown promising results, and their usage is limited due to their undesirable side effects. In connection with the above-described reports, AAL extract could be a promising and effective treatment against neurodegenerative diseases, including AD.

## 4. Materials and Methods

### 4.1. Preparation of Ethanol Extract from AAL

AAL was provided by Sunny Farm (Jeju, South Korea). Dried AAL (2.7 kg) was extracted twice with 60 L ethanol using an electric extractor (COSMOS-660, Kyungseo Machine Co., Incheon, Korea) for 3 h each time. The filtered extract solution was evaporated and freeze-dried to yield 868.03 g (yield = 32.15%) of powdered extracts. A sample specimen (SCD-A-111) was kept at the Clinical Medicine Division, Korea Institute of Oriental Medicine.

### 4.2. Reagents and Chemicals

Rutin and isoquercitrin, as standard components, were obtained from ChemFaces Biochemical Co., Ltd. (Wuhan, China), and these standard components were purified to ≥98.0% by high-performance liquid chromatography (HPLC) analysis. The HPLC-grade water and acetonitrile were obtained from J. T. Baker Chemical Co. (Phillipsburg, NJ, USA), and the trifluoroacetic acid (TFA) was obtained from Sigma-Aldrich (St. Louis, MO, USA).

### 4.3. Preparation of Sample and Standard Solutions for HPLC Analysis

The powdered AAL extract was weighed at 10 mg/mL and dissolved in 90% aqueous methanol. The sample solution was passed through a syringe filter (0.45 µm) for HPLC analysis. To make standard solutions of the two compounds, each compound was weighed accurately and dissolved at a concentration of 1.0 mg/mL in methanol, and these stock solutions were mixed and diluted before HPLC analysis.

### 4.4. Chromatographic Conditions

HPLC analysis was used Waters Alliance e2695 HPLC system (Waters Corp., Milford, MA, USA) equipped with a pump, degasser, column oven, automatic sample injector, and photodiode array (PDA) detector (#2998; Waters Corp.). Chromatographic separation for the two standard compounds was carried out with a Luna C18 analytical column (250 × 4.6 mm, 5 µm, Phenomenex, Torrance, CA, USA) maintained at 40 °C. The mobile phases consisted of 0.1% (*v*/*v*) aqueous TFA (A) and acetonitrile (B). The gradient conditions were as follows; 10% B for 0–10 min, 10–30% B for 10–40 min, and 100% B for 40–50 min. The flow rate was 1.0 mL/min, and the injection volume was 10 µL. The data were acquired and processed using Empower software (version 3; Waters Corp).

### 4.5. Animal and Drug Treatments

The experiments were performed according to the National Institutes of Health (NIH) Guide for the Care and Use of Laboratory Animals and approved by the Korea Institute of Oriental Medicine Institutional Animal Care and Use Committee (IACUC Approval No.17-044, Approval date 29, March, 2017). Seven-week-old male ICR mice were purchased from the Daehan Biolink (Cheongju, Korea) and acclimated for 1 week prior to the study with standard food and water supplied ad libitum in individual acryl cages. All mice were housed under controlled conditions (12 h light/dark cycle; temperature: 22 ± 2 °C; 55% humidity). The study began when the ICR mice were 8 weeks old (weight: ~35 g), and the mice were monitored for 3 weeks. Memory deficit was induced by a single injection of SCO (1 mg/kg, i.p.). Age-matched control mice received an equal volume of vehicle (phosphate buffered saline, PBS). Fifty-eight male ICR mice were randomly divided to one of 6 groups (*n* = 8). AAL extract was dissolved in distilled water (DW) to concentrations of 50, 100, and 200 mg/kg and administered daily by gastric gavage; the other groups were given TAC (USP, Rockville, MD, USA) as a positive control (10 mg/kg). All mice underwent behavioral tests from the 15th to the 20th day. All mice were injected with SCO within 60 min after oral administration of AAL extract or TAC solution except for mice in the normal control group, which received saline (i.p.) 30 min after treatment with PBS. The behavioral tests were conducted 30 min after SCO injection. All efforts were made to minimize animal suffering. The animal equivalent dosage was determined from the human equivalent dose. We considered that the usual dosage of the plant is approximately 20–80 g/70 kg/day of the raw plant for adult human. The calculated animal dose range was from 40 to 250 mg/kg in mice. The AAL dose for this study was determined 50, 100, or 200 mg/kg per day. Animal experiments were performed under nontoxic concentration of AAL. This animal study procedures were performed according to ARRIVE guideline.

### 4.6. Brain Section and Tissue Preparation

All mice were sacrificed under anesthesia at the end of the experimental period and the hippocampal and cortex tissues were immediately isolated on ice and stored at −80 °C for further analysis. Three mouse brains from each group were perfused intracardially with PBS (pH 7.4) and fixed in 4% paraformaldehyde.

### 4.7. Western Blot Analysis

Hippocampal and cortical brain tissues were homogenized in RIPA buffer (pH 7.5) with phosphatase and protease inhibitors (Pierce Biotechnology, Rockford, IL, USA). The proteins were separated on 4–20% gradient polyacrylamide gels and then transferred on PVDF membranes. Blocked PVDF membranes with 5% nonfat milk were washed with TBST solution for nonspecific binding. The blocked membranes were incubated with rabbit anti-total CREB, pCREB, ChAT, total Akt, pAkt, cleaved caspase-3, Bax, Bcl2 (Cell Signaling Technology, Danvers, MA, USA, 1:2000 dilution), and mouse anti-ß-actin (Santa Cruz Biotechnology Inc., Dallas, TX, USA, 1:3000 dilution). Washed membranes were incubated with secondary antibodies with conjugated horseradish peroxidase and immunoblot bands were developed using supersignal ECL solution (Amersham Bioscience, Piscataway, NJ, USA). Protein levels were detected by analyzing the captured signals using a ChemiDox imaging analyzer (Las-4000 MINI, Fuji photo, Tokyo, Japan).

### 4.8. Measurement of ACh Level and AChE Activity Assay

ACh levels and AChE activity in hippocampal and cortical tissues were measured using respective assay kits (US Biomax Inc, Denwood, MD, USA) in accordance with the manufacturer’s protocol. Absorbance of mixture was measured at 570 nm using a spectrophotometer (Benchmark Plus, Bio-Rad, Hercules, CA, USA).

### 4.9. Nissl and TUNEL Staining

After 3 weeks of AAL extract treatment, fixed brain tissues with 4% paraformaldehyde were embedded in paraffin, and 4-μm-thick sections were prepared. Staining was performed as previously described [56]. Deparaffinized and hydrated slides were dipped in 1% Nissl stain solution (cresyl violet) for 1 min and washed with tap water. TUNEL staining was performed with an in situ cell death detection AP kit (Roche Diagnostics, Mannheim, Germany) according to the manual’s instruction. Apoptotic cells were stained with a color solution containing nitroblue tetrazolium and 5-bromo-4-chloro-3-indolylphosphate (NBT/BCIP, Roche Diagnostics). Stained sections were then visualized using an Olympus DP71 (Tokyo, Japan) at 400 magnification. The images were analyzed using ImageJ software (Java-based image processing program, NIH).

### 4.10. Passive Avoidance Test (PAT)

The PAT, conducted using two identical compartments (Electronic shock generator, Jeungdo Bio & Plant Co. Ltd., Seoul, Korea) consisted of lighted and darkened compartment with a door in between compartments and electrifiable grid floor. During the acquisition phase, mice were placed in the lighted compartment for familiarization for 25 s and then crossed to the darkened compartment. The door was opened and then received a mild electrical shock (0.3 mA, 3 s). In retention phase, mice were again placed in the lighted compartment. The latency time of the darkened compartment required the mice to remain in lighted compartment was recorded as the retention time. If the mice did not enter the darkened compartment within 5 min, the latency time was recorded as 300 s. No physiological defects (i.e., motor deficits) or intrinsic cognitive impairments were observed in any of the mouse groups prior to treatment with SCO.

### 4.11. Y-Maze Test

The Y-maze (length: 35 cm; height: 15 cm; width: 7 cm) tests were positioned at equal angles three arms from one another. Each mouse was placed in one arm and allowed to freely explore the maze for 8 min. The number of spontaneous alternations was recorded using a tracking system (EthoVision XT, Noldus Information Tech, Wageningen, The Netherlands). Spontaneous alternation (%) was considered when mice entered into all three arms without repetition and the rate of spontaneous alternation was as follows; % alternation = [(number of alternations)/(total number of arm entries-2)] × 100.

### 4.12. Measurement of Reactive Oxygen Species (ROS) in Hippocampus

The total free radical presence in the homogenized hippocampus brain tissues was determined using an ROS/reactive nitrogen species (RNS) free radical activity assay kit (Cell Biolabs, Inc., San Diego, CA, USA) according to the manufacturer’s protocol. Fifty microliters of sample solution, hydrogen peroxide solution, or 2′, 7′-dichlorodihydrofluorescein (DCF) solution was added to each well of a 96-well plate, and 50 μL of catalyst was added to each well, followed by incubation for 5 min at room temperature. Then, 100 μL of prepared dichlorodihydrofluorescin (DCFH) solution was added to each well. The plate was covered and incubated at room temperature for 45 min. The fluorescence intensity of the resulting solution was measured at 480/530 nm excitation and emission with a SpectraMax multimode detection platform fluorometer (Molecular Devices, Sunnyvale, CA, USA).

### 4.13. Statistical Analysis

All data were expressed as the mean ± SEM and statistical analyses were evaluated via a one-way analysis of variance (ANOVA) followed by an unpaired Student’s *t*-test or Tukey’s multiple comparison test. All experiments were performed individually at least three times. GraphPad Prism 8.0 software program (Graph pad, San Diego, CA, USA) was used for all analyses. Difference at *p* < 0.05 was considered to be statistical significance.

## 5. Conclusions

The effects of AAL on improving memory loss resulted from ameliorating cholinergic system dysfunction, which could lead to the progression of neurodegenerative disorders, and preventing apoptotic activity, which would otherwise promote neuronal damage due to oxidative stress in the brain. Moreover, AAL extract treatment exerted memory-enhancing effects via inhibition of the BDNF/pCREB and Akt signaling pathways in SCO-induced cognitive deficit mice. Taken together, our findings suggest that AAL extract—a natural product—could be potentially used to develop a novel drug for the treatment of AD.

## Abbreviations

ACh	Acetylcholine
AChE	Acetylcholinesterase
pAkt	Phophorylated protein kinase B (Akt)
PBS	Phosphate-buffered saline
Bcl2	B-cell lymphoma 2
Bax	Bcl2-associated X protein
pCREB	Phosphorylated cAMP response elements binding protein
BDNF	Brain derived neurotrophic factor
ChAT	choline acetyltransferase
HPLC	High-performance liquid chromatography
TFA	Trifluoroacetic acid
ABTS	2,2′-azino-bis (3-ethylbenzothiazoline-6-sulfonic acid)
DPPH	2,2diphenyl-1-picrylhydrazyl
DCFH	dichlorodihydrofluorescin
DCF	2′, 7′-dichlorodihydrofluorescein
RIPA	Radioimmunoprecipitation assay buffer
ROS	reactive oxygen species
TUNEL	Terminal deoxynucleotidyl transferase dUTP nick end labeling
